# Selective Removal of the Genotoxic Compound 2-Aminopyridine in Water using Molecularly Imprinted Polymers Based on Magnetic Chitosan and β-Cyclodextrin

**DOI:** 10.3390/ijerph14090991

**Published:** 2017-08-31

**Authors:** Wei Zhang, Zhiliang Zhu, Hua Zhang, Yanling Qiu

**Affiliations:** 1 State Key Laboratory of Pollution Control and Resource Reuse, Tongji University, Shanghai 200092, China; zhangw21@sina.com (W.Z.); zhhua@tongji.edu.cn (H.Z.); 2 Key Laboratory of Yangtze River Water Environment, Ministry of Education, Tongji University, Shanghai 200092, China; ylqiu@tongji.edu.cn

**Keywords:** aminopyridine, magnetic molecularly imprinted polymer, selective adsorption, chitosan, β-cyclodextrin, genotoxic compounds

## Abstract

To develop efficient materials with enhanced adsorption and selectivity for genotoxic 2-aminopyridine in water, based on magnetic chitosan (CTs) and β-cyclodextrin (β-CD), the magnetic molecularly imprinted polymers (MMIPs) of Fe_3_O_4_-CTs@MIP and Fe_3_O_4_-MAH-β-CD@MIP were synthesized by a molecular imprinting technique using 2-aminopyridine as a template. The selective adsorption experiments for 2-aminopyridine were performed by four analogues including pyridine, aniline, 2-amino-5-chloropyridine and phenylenediamine. Results showed the target 2-aminopyridine could be selectively adsorbed and quickly separated by the synthesized MMIPs in the presence of the above structural analogues. The coexisting ions including Na^+^, K^+^, Mg^2+^, Ca^2+^, Cl^−^ and SO_4_^2−^ showed little effect on the adsorption of 2-aminopyridine. The maximum adsorption capacity of 2-aminopyridine on Fe_3_O_4_-CTs@MIP and Fe_3_O_4_-MAH-β-CD@MIP was 39.2 mg·g^−1^ and 46.5 mg·g^−1^, respectively, which is much higher than values in previous reports. The comparison result with commercial activated carbon showed the obtained MMIPs had higher adsorption ability and selectivity for 2-aminopyridine. In addition, the synthesized MMIPs exhibited excellent performance of regeneration, which was used at least five times with little adsorption capacity loss. Therefore, the synthesized MMIPs are potential effective materials in applications for selective removal and analysis of the genotoxic compound aminopyridine from environmental water.

## 1. Introduction

Pyridine and its derivatives aminopyridines are widely used as starting materials and key intermediates in the production of pharmaceuticals, pesticides, dyes, and rubber [1]. The release of aminopyridines into the environment through various waste streams has attracted more and more attention, due to their high toxicities, carcinogenic potential and hazardous effect on ecosystem and human health [1]. Because of their relatively high solubility in water, aminopyridines can easily permeate through soil and contaminate groundwater and are difficult to degrade under aerobic and anaerobic conditions. Therefore, these substances were listed as priority pollutants by U.S. Environmental Protection Agency (U.S. EPA). Among the three monoaminopyridines, 2-aminopyridine is the most widespread in the production of various drugs, especially antihistamines and piroxicam. A previous investigation indicated that 2-aminopyridine is mutagenic, carcinogenic, and biodegradation-resistant when exposed in the environment [2]. Hence, it is necessary to decontaminate 2-aminopyridine-polluted water prior to discharge into the environment. As for the current situation, it is urgent to develop environmentally friendly and cost-effective techniques for removal of contaminants such as aminopyridines in water. So far, several techniques and methods have been reported and are available for treatment of the aminopyridines such as advanced oxidation [3], electrochemical [4] and adsorption [5]. Among these techniques, selective separation by molecular imprinted polymers (MIPs) has attracted more attention because of its advantages such as high selectivity, good stability, low cost as well as easy operation [6,7,8,9]. Molecularly imprinted polymers are typically obtained by polymerization of targeted template molecules with functional monomers by covalent or non-covalent interactions. Specific binding sites can be created within the three-dimensional polymer after removing templates, which provide great binding affinity for the template molecules [10,11,12]. However, the conventional MIPs have several drawbacks including slow mass transfer, poor site accessibility and low binding capacity [13]. To overcome the above shortcomings, the imprinting techniques have been developed on the surface of supporting materials, such as chitosan, carbon nanotubes, silica particles, titanium dioxide and cyclodextrins, etc. [14,15,16,17,18,19].

Among these surface-imprinted polymers, chitosan- (CTs) and cyclodextrin (CD)-based polymers have received much attention for the removal of contaminants in recent years [20,21,22]. Chitosan as a useful commercial polymer contains numerous amino and hydroxyl functional groups, which can provide the flexibility for structural modifications and molecularly imprinted polymers preparation [23]. β-cyclodextrin (β-CD) with 7 d-glucopyranonsyl residue form a ring by α-1,4-glucosidic bonds, including a hydrophobic inner cavity and a hydrophilic outer surface. Due to their special structure, various targeted molecules can be easily bonded into β-CD specific cavities and react with the hydroxyl groups located on the surface, to form stable inclusion complexes through intermolecular interactions during the imprinting process [24]. In addition, effective and quick separation of the adsorbents from contaminated water is of great importance in wastewater treatment. Magnetic separation technique is a good method; it is quick and convenient to separate magnetic polymers from a complex matrix by an external magnetic field [25,26].

So far, the investigations on imprinted polymers of toxic aminopyridines for selective adsorption from aqueous solutions are still limited. To improve their absorption capacity, selectivity and enhance the removal efficiency, the design and functionalization of novel adsorbents still need to be explored. Zhou et al. [13] reported a molecularly imprinted polymer for the recognition of 2-aminopyridine synthesized by bulk polymerization. The prepared monoliths need to be ground and sieved to the appropriate particle size, which is time-consuming and may also be detrimental to some of the binding sites. Thus, the adsorption capacity and selectivity are inevitably reduced. Temperature-responsive imprinted polymer hydrogels were synthesized by Liu et al. [27] for the adsorption of 4-aminopyridine. The imprinted hydrogels can adsorb and release reversible 4-aminopyridine by changing the temperature. However, the reported imprinted polymers of aminopyridine showed low adsorption capacity and also difficulty in quick separation. The objective of this work is to develop more efficient imprinted polymer materials, which have higher adsorption capacity and can be easily separated from water. In this work, two novel magnetic MIPs based on chitosan and β-CD were synthesized using the molecular imprinting technique. Maleic anhydride-modified β-CD containing a vinyl functional group was used to copolymerize with methacrylic acid (MAA) to form molecularly imprinted polymers with the target template through electrostatic and hydrogen bond interaction cooperatively. The magnetic MIPs with easy separation performance were obtained after removal of the template. The adsorption behavior of the synthesized MMIPs toward 2-aminopyridine, which includes isotherm, kinetics, thermodynamics, selective recognition and the effect of solution pH, was investigated in detail. The possible mechanisms were also discussed. Additionally, the results were compared with commercial activated carbon for the removal of 2-aminopyridine, through simulating contaminated environmental water samples.

## 2. Materials and Methods

### 2.1. Reagents and Chemicals

Chemicals including pyridine, 2-aminopyridine (2-AP), aniline, 2-amino-5-chloropyridine (2-A-5-CP), phenylenediamine, maleic anhydride (MAH), oleic acid were purchased from TCI Development Co., Ltd. (Shanghai, China). Monomer methacrylic acid (MAA) and ethyleneglycol dimethacrylate (EGDMA) were obtained from Sigma-Aldrich. 2, 2’-azobis (2-methylpropionitrile) (AIBN, initiator) was purchased from J&K Technology Limited (Beijing, China). FeCl_3_·6H_2_O, FeCl_2_·4H_2_O, polyvinylpyrrolidone (PVP), ammonium hydroxide (NH_3_·H_2_O) and chitosan (CTs) with 95% deacetylation and an average molecular weight of 1 × 10^5^ g·mol^−1^ were obtained from Sinopharm Chemical Reagent (Shanghai, China). β-cyclodextrin (β-CD) was supplied by TCI and recrystallized for purification. *N*,*N*-Dimethylformamide, acetonitrile, chloroform, toluene, acetic acid and other reagents were AR or HPLC grade and were supplied by Fisher Scientific (Shanghai, China).

### 2.2. Synthesis of Magnetic Chitosan Imprinted Polymer

#### 2.2.1. Synthesis of Fe_3_O_4_-Chitosan Particles

The Fe_3_O_4_-chitosan particles were prepared using a one-step modifying process [28]. A typical procedure was described as follows. An aqueous solution (20 mL) containing FeCl_3_·6H_2_O (0.7 g) and FeCl_2_·4H_2_O (0.3 g) was dispersed ultrasonically into 100 mL of chitosan (0.5 g) solution with 0.5% (*v/v*) acetic acid under nitrogen atmosphere. After being stirred for 1 h at 60 °C, the solution was added to 15 mL of NH_3_·H_2_O, drop by drop, and vigorously stirred for another 30 min. Finally, Fe_3_O_4_-chitosan magnetic particles were collected by a magnet and washed thoroughly with purified water. The products were then dried overnight at 50 °C in a vacuum oven.

#### 2.2.2. Synthesis of Fe_3_O_4_-CTs@MIP

**Figure 1:**
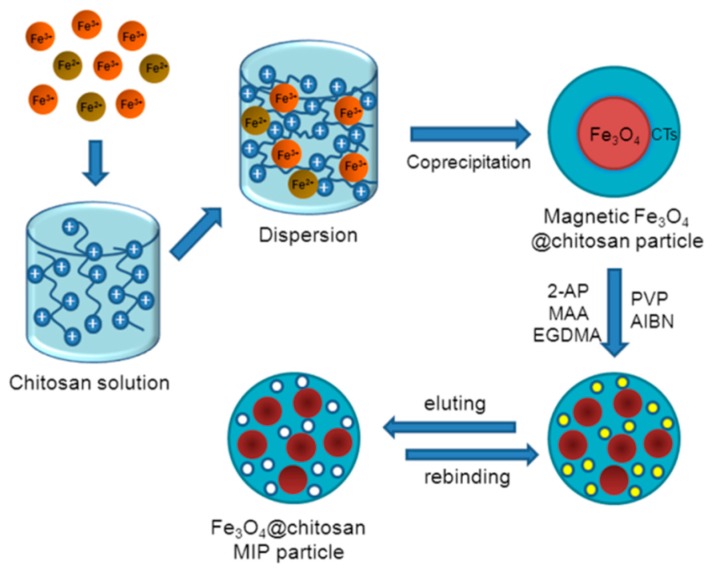
Scheme of preparation of Fe_3_O_4_-CTs@MIP.

The Fe_3_O_4_-CTs@MIP was synthesized by the molecular imprinted method as described in Figure 1. Typically, 2-aminopyridine (1 mmol) was dissolved in 10 mL of acetonitrile: toluene (75:25, *v/v*) in a three-necked flask. Then, MAA (4 mmol) was added and stirred for 1 h. Subsequently, 100 mL of acetonitrile: toluene (75:25, *v/v*) containing 300 mg of Fe_3_O_4_-chitosan particles and 1.0 mL oleic acid were added and continuously stirred under ultrasonication for 20 min. The dispersant PVP (0.4 g), EGDMA (20 mmol) and AIBN (90 mg) were added successively to the mixture. The final mixture was purged with nitrogen while the reaction temperature increased to 60 °C and then maintained at 60 °C for 24 h. After polymerization, the Fe_3_O_4_-CTs@MIP was collected with an external magnet and Soxhlet extracted with methanol: acetic acid (9:1, *v/v*) until the template could not be detected in the filtrate. The obtained polymer was dried under vacuum overnight at 50 °C. Additionally, a magnetic non-imprinted polymer (Fe_3_O_4_-CTs@NIP) was synthesized with a similar procedure but without adding the template. To establish the reproducibility of the Fe_3_O_4_-CTs@MIP preparation protocols, three batches of polymer strictly following the protocol outlined above were conducted.

### 2.3. Synthesis of Magnetic β-Cyclodextrin Imprinted Polymer

#### 2.3.1. Synthesis of MAH-β-CD

Typically, β-CD (5.68 g) and maleic anhydride (4.9 g) were added sequentially and dissolved in 30 mL DMF. The mixture was gradually heated to 80 °C and then maintained for 10 h with vigorous stirring. After termination of the reaction, the mixture was naturally cooled to room temperature and then 30 mL of trichloromethane was slowly added. The obtained white precipitate was filtrated and rinsed several times with sufficient acetone. Finally, the product was dried under vacuum at room temperature for one day and then kept at 80 °C for 3 days.

#### 2.3.2. Synthesis of MAH-β-CD Polymer Coated Magnetic Particles

Epichlorohydrin (4 mL) was dropped into sodium hydroxide solution (20 mL, 10% (*w/v*)) containing MAH-β-CD (2 g) and then stirred vigorously for 8 h. After the mixture solution became clear, epichlorohydrin (2 mL) was added again, and the solution was stirred overnight. The solution was concentrated and precipitated by adding cold ethanol. A fine precipitate was obtained by crushing the gummy precipitate several times with ethanol. The product was continually rinsed with ethanol and acetone and dried under strong vacuum overnight.

MAH-β-CD polymer-coated magnetic particles were prepared by modifying the method in Ref [29]. In brief, FeCl_2_·4H_2_O (0.86 g), FeCl_3_·6H_2_O (2.36 g) and MAH-β-CD polymer (1.5 g) were dispersed into 40 mL of purified water under an N_2_ atmosphere with vigorous stirring. Then, 5 mL of NH_3_·H_2_O was added after the solution was heated to 90 °C and maintained for 1 h. Fe_3_O_4_-MAH-β-CD magnetic particles were collected by a magnet and rinsed thoroughly with purified water. The product was then dried in a vacuum oven at 50 °C for 24 h.

#### 2.3.3. Synthesis of Fe_3_O_4_-MAH-β-CD@MIP

**Figure 2:**
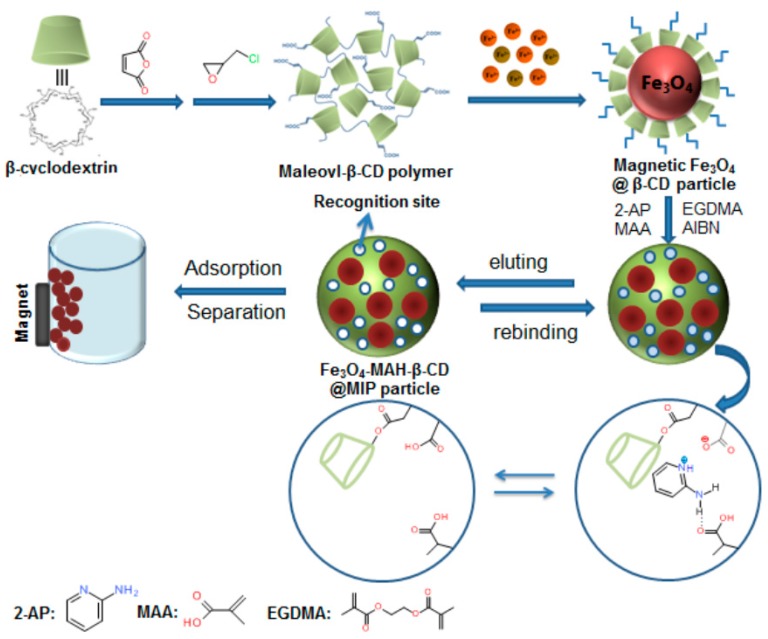
Scheme of preparation of Fe_3_O_4_-MAH-β-CD@MIP and application for removal of 2-aminopyridine with external magnetic field.

The Fe_3_O_4_-MAH-β-CD@MIP was prepared by the molecular imprinted technique as described in Figure 2. Typically, 2-aminopyridine (0.3 mmol) and MAA (1.2 mmol) were added and dissolved in 20 mL DMF. Then, Fe_3_O_4_-MAH-β-CD particles (340 mg) were dispersed into the above solution, with ultrasonication for 20 min. EGDMA (6 mmol) and AIBN were added sequentially to the mixture under nitrogen atmosphere and reacted at 60 °C for 24 h. Finally, the product was Soxhlet extracted with methanol:acetic acid (9:1, *v/v*) and washed with purified water to ensure no more template could be detected. The obtained polymer was dried under vacuum overnight. For comparison, Fe_3_O_4_-MAH-β-CD non-imprinted polymer (Fe_3_O_4_-MAH-β-CD@NIP) without 2-aminopyridine template was also prepared for evaluation of the imprinting efficiency. In addition, three batches of Fe_3_O_4_-MAH-β-CD@MIP were conducted strictly following the protocol outlined above for the evaluation of reproducibility of preparation protocols.

### 2.4. Characterization

The morphology of the MMIPs was analyzed by a scanning electron microscope (SEM) (FEI, Nova NanoSEM 450, Hillsboro, OR, USA) in secondary electron mode, with an acceleration voltage of 10 kV. The samples were coated with a thin film of gold to enable SEM imaging. The magnetic properties (saturation magnetization and coercivity) were measured using a Lakeshore 7407 vibrating sample magnetometer. X-ray powder diffraction (XRPD) patterns were collected with a PANalytical Empyrean diffractometer using monochromatic Cu Kα radiation (λ = 0.154 nm). The applied voltage and current were set to 45 kV and 40 mA, respectively. The pattern was scanned over a 2θ angle range from 20° to 70°. FT-IR spectroscopy was measured using a thermo Nicolet iS10 FT-IR spectrometer. ^1^H NMR and ^13^C NMR spectra were obtained from a Bruker Avance III-400M NMR spectrometer with D_2_O as the solvent. Thermo-gravimetric analysis (TGA) was carried out using a Q5000 thermogravimetric analyzer (TA Instruments, New Castle, DE, USA) over a temperature range of 30–700 °C. The scan rate was set to 10 °C·min^−1^ with dry nitrogen gas.

### 2.5. Adsorption Experiments

Batch adsorption experiments were conducted by adding 20 mg of adsorbents into 10 mL of 2-aminopyridine solution with initial concentrations (C_0_) ranging from 0.01 mg·mL^−1^ to 0.6 mg·mL^−1^ at 25 °C. After being shaken for 24 h, the adsorbents were separated with an external magnetic field, and the concentration of equilibrium solutions (C_e_) were determined by UPLC. The equilibrium adsorption capacity (Q_e_, mg·g^−1^) was calculated:

(1) $$Q_{e}=\frac{(C_{0}-C_{e})V}{m}$$

where V (mL) is the volume of the initial solution and m (g) is the weight of the adsorbent.

The effect of the initial solution pH on 2-aminopyridine adsorption was investigated. The initial pH of the 2-aminopyridine solution was adjusted to the range of 2–12 by adding acid or alkaline solutions. For the kinetics study, the suspensions were shaken at 25 °C and sampled at different intervals of time in 2-aminopyridine solutions with the initial concentration of 0.1 mg·mL^−1^ at optimal pH value. The pseudo-first-order kinetic model, the pseudo-second-order kinetic model, and the intraparticle diffusion model were used to describe the adsorption kinetics. The adsorption thermodynamic constants of ΔH, ΔG and ΔS were also calculated.

### 2.6. Selective Adsorption

Experiments of selective recognition ability were performed with 2-aminopyridine (2-AP) and four analogous compounds including pyridine, aniline, 2-amino-5-chloropyridine (2-A-5-CP), phenylenediamine. Adsorbents (20 mg) were added into a 10 mL aqueous solution containing 0.1 mg·mL^−1^ of each above-mentioned compound. After shaking for 24 h, the supernatants were filtered through a 0.22 μm filter for UPLC analysis.

### 2.7. Influence of Coexistent Ions

The adsorption performance and selective ability of MMIPs and MNIPs for 2-aminopyridine in the presence of coexisting ions such as Na^+^, K^+^, Mg^2+^, Ca^2+^, Cl^−^ and SO_4_^2−^ in water were investigated. The concentration of 2-aminopyridine varied from 0.001 mg·mL^−1^ to 0.01 mg·mL^−1^, and the initial concentrations of Na^+^, K^+^, Mg^2+^, Ca^2+^, Cl^−^ and SO_4_^2−^ were 1.149 mg·mL^−1^, 0.039 mg·mL^−1^, 0.122 mg·mL^−1^, 0.04 mg·mL^−1^, 1.882 mg·mL^−1^ and 0.48 mg·mL^−1^, respectively.

### 2.8. The Contrast of MMIPs and Activated Carbon in Selective Adsorption Performance

Spiked water samples (10 mL) contained 0.1 mg·mL^−1^ of 2-aminopyridine, and analogous using 20 mg synthesized MMIPs or granular active carbon (GAC) were applied successively. After being shaken for 4 h at 25 °C, the supernatants were centrifuged and filtered. The concentrations of free 2-aminopyridine and analogous in the filtrate were determined by UPLC.

### 2.9. Regeneration and Reusable Studies

Synthesized MMIPs (20 mg) were added to 10 mL of 0.1 mg·mL^−1^ 2-aminopyridine spiked water samples. After being shaken for 4 h at 25 °C, the mixture was centrifuged and filtered. Then, the concentration of filtrate was analyzed by UPLC. The recovered MMIPs were regenerated with 5 mL of methanol/acetic acid (90:10, *v/v*). After drying in vacuum, it was reused in the next cycle of sorption experiments.

### 2.10. Removal of 2-Aminopyridine from Different Water Samples

Three types of water samples including deionized water, tap water and river water were used for evaluation of the removal performance of 2-aminopyridine. The deionized water was prepared from a Millipore system, and tap water was obtained directly from the laboratory. River water was collected from the Huangpu River in Shanghai and stored under cool and dark conditions. These water samples were filtered through a 0.22 μm filter prior to use. 2-aminopyridine was not detected in the original water samples by UPLC. MMIPs (20 mg) were suspended in 10 mL of water spiked with 0.01 mg·mL^−1^ 2-aminopyridine at room temperature for 2 h under shaking, then centrifuged and filtered by 0.22 μm filter for UPLC analysis.

## 3. Results and Discussion

### 3.1. NMR Analysis of MAH-β-CD

**Figure 3:**
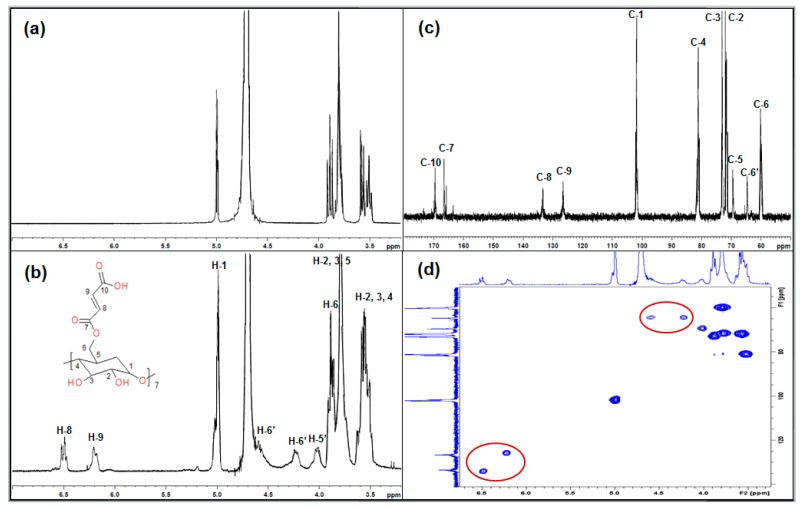
^1^NMR spectra of β-CD in D_2_O (**a**); NMR spectra of MAH-β-CD in D_2_O. ^1^H spectrum (**b**); ^13^C spectrum (**c**); 2D HSQC ^13^C–^1^H plot (**d**).

The ^1^H NMR spectra of β-CD and MAH-β-CD are shown in Figure 3a,b. The ^1^H NMR assignments of β-CD were as follows: ^1^H NMR (D_2_O): δ = 4.99(H_1_); δ = 3.49–3.91(H_2_, H_3_, H_4_, H_5_, H_6_), and that of β-CD-g-MAH were: ^1^H NMR (D_2_O): δ = 6.52, 6.20(H_8_, H_9_); δ = 4.99(H_1_); δ = 3.48–3.91(H_2_, H_3_, H_4_, H_5_, H_6_); δ = 4.57, 4.24(H_6′_); δ = 4.03(H_5′_).

Comparing the ^1^H NMR of β-CD with MAH-β-CD, new peaks δ = 6.52, 6.20 (H_8_, H_9_); δ = 4.57, 4.24 (H_6′_), δ = 4.03(H_5′_) were observed in MAH-β-CD. The two-dimensional (2D) HSQC ^13^C–^1^H plot (Figure 3d) provides a detailed interpretation of these protons: δ_H_ 6.52 was correlated with δ_C_ 133.37, δ_H_ 6.20 was correlated with δ_C_ 126.37; therefore, δ_H_ 6.52 and δ_H_ 6.20 were attributed to alkene protons, δ_C_ 133.37 and δ_C_ 126.37 (Figure 3c) were due to the carbon-carbon double bond of maleic anhydride unit. This result indicated that β-CD reacted with MAH. δ_H_ 4.57 and δ_H_ 4.24 were correlated with the same carbon δ_C_ 64.64; therefore, the two protons belong to a methylene group, and the H-6 chemical shift change further proved that the reaction position was 6-OH. Therefore, the functionalized maleic anhydride β-CD was synthesized successfully.

### 3.2. Characterization of the Magnetic Fe_3_O_4_-CTs@MIP and Fe_3_O_4_-MAH-β-CD@MIP

**Figure 4:**
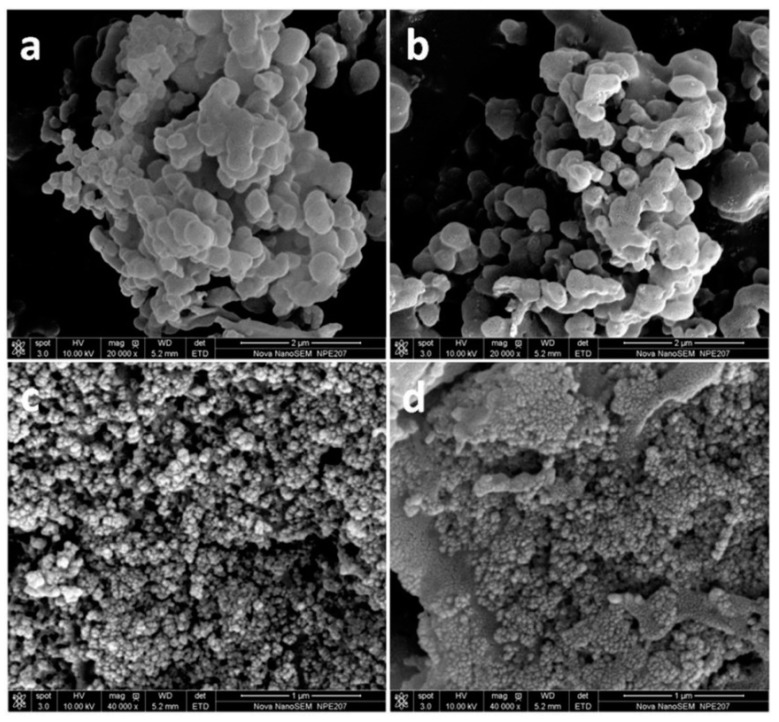
SEM images of Fe_3_O_4_-CTs@MIP (**a**), Fe_3_O_4_-CTs@NIP (**b**), Fe_3_O_4_-MAH-β-CD@MIP (**c**) and Fe_3_O_4_-MAH-β-CD@NIP (**d**).

The surface morphologies of Fe_3_O_4_-CTs@MIP, Fe_3_O_4_-CTs@NIP, Fe_3_O_4_-MAH-β-CD@MIP and Fe_3_O_4_-MAH-β-CD@NIP were characterized by SEM. Figure 4a,b showed no significant differences between Fe_3_O_4_-CTs@MIP and Fe_3_O_4_-CTs@NIP on SEM images. It can be found the morphologies with the agglomerate consisted of several spherical magnetite particles. The SEM of the prepared Fe_3_O_4_-MAH-β-CD@MIP and Fe_3_O_4_-MAH-β-CD@NIP is presented in Figure 4c,d. The Fe_3_O_4_-MAH-β-CD@MIP and Fe_3_O_4_-MAH-β-CD@NIP showed appreciable differences in morphology. The Fe_3_O_4_-MAH-β-CD@NIP crosslinking is more compact, whereas the Fe_3_O_4_-MAH-β-CD@MIP exhibited more rough and porous structures than that of Fe_3_O_4_-MAH-β-CD@NIP, indicating that the removal of template molecules resulted in possible recognition sites in Fe_3_O_4_-MAH-β-CD@MIP. It is favorable for the formation of dimensional multiple-point binding sites. The particle size of Fe_3_O_4_-MAH-β-CD@MIP was also uniform.

The FT-IR analysis results are shown in Figure S1. In Figure S1a, a strong and wide band around 3282 cm^−1^ was contributed by the absorption of hydroxyl; C-O stretching vibration at 1028 cm^−1^ contributed to the linkage of hydroxylgroups with β-CD. The peak at 1718 cm^−1^, assigned to C=O stretching vibration, and the peak at 1640 cm^−1^, assigned to C=C stretching vibration, were observed in MAH-β-CD, which means that β-CD modified with vinyl carboxylic acid groups was synthesized successfully, which was consistent with previous reports [30].

Figure S1b shows the FT-IR spectra of chitosan, Fe_3_O_4_ particle, Fe_3_O_4_-CTs, Fe_3_O_4_-CTs@MIP and Fe_3_O_4_-CTs@NIP. The peak at 590 cm^−1^ was assigned to Fe-O stretching vibration, which proved that the Fe_3_O_4_ was successfully coated onto the polymers. For chitosan, the broad band around 3400 cm^−1^ was attributed to –OH and –NH_2_ stretching vibration. The weak band at 2920 and 2869 cm^−1^ was the characteristic absorbance peak of –CH_2_. The absorption peak at 1590 cm^−1^ was assigned to –NH_2_ bending vibration and the primary –OH alcohol bond. Additionally, the absorption peaks of symmetric stretching of C-O-C appeared at 1062 cm^−1^ and 1022 cm^−1^. Compared with the spectra of chitosan and Fe_3_O_4_, Fe_3_O_4_-CTs exhibited a typical peak of C-O-C at 1067 cm^−1^ as well as a peak at 2918 cm^−1^, which might be attributed to the CH_2_ group in chitosan. In addition, the typical strong adsorption peak at 1728 cm^−1^ was exhibited in Fe_3_O_4_-CTs@MIP and Fe_3_O_4_-CTs@NIP, which could be clearly attributed to the C=O stretching vibration of EGDMA and MMA. The O–H band vibration was 3432 cm^−1^ of Fe_3_O_4_-CTs@MIP, demonstrating the existence of a hydrogen bond in MMIP.

Figure S1c shows the FT-IR spectra of the Fe_3_O_4_ particle, Fe_3_O_4_-MAH-β-CD, Fe_3_O_4_-MAH-β-CD@MIP and Fe_3_O_4_-MAH-β-CD@NIP. The presence of the peak at 1723 cm^−1^ was attributed to C=O stretching, which confirmed that the carboxy group was incorporated into MAH-β-CD polymer. The peak at around 3400 cm^−1^ corresponding to the O−H stretching vibration of hydroxyl groups from β-CD appeared in both two imprinted polymers. The peak at 1729 cm^−1^ was assigned to the stretching vibration of the carbonyl from the functional monomer MAA as well as EGDMA. The peak at 590 cm^−1^, assigned to Fe-O bond vibration of Fe_3_O_4_, was observed in all polymers.

**Figure 5:**
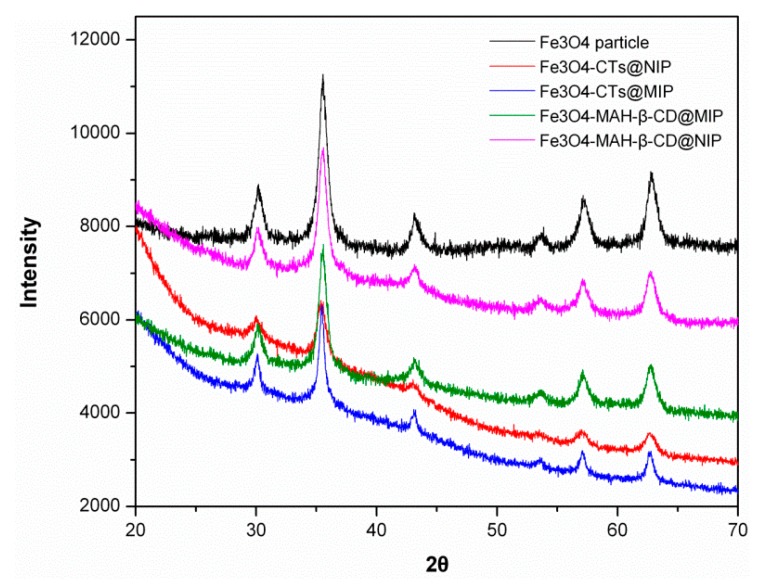
XRPD patterns of Fe_3_O_4_, Fe_3_O_4_-CTs@MIP, Fe_3_O_4_-CTs@NIP, Fe_3_O_4_-MAH-β-CD@MIP and Fe_3_O_4_-MAH-β-CD@NIP.

Figure 5 presents the XRPD patterns of the Fe_3_O_4_ particle, Fe_3_O_4_-CTs@MIP, Fe_3_O_4_-CTs@NIP, Fe_3_O_4_-MAH-β-CD@MIP and Fe_3_O_4_-MAH-β-CD@NIP. Six characteristic diffraction peaks at 2θ = 30.2°, 35.5°, 43.2°, 53.5°, 57.1° and 62.8° were clearly observed in the 2θ range of 20°–70° for all the magnetic samples, which matched well with the JCPDS card No. 89-3854 for the cubic structure of Fe_3_O_4_. These characteristic diffraction peaks correspond to (220) (311) (400) (422) (511), and (440) reflection planes of the Fe_3_O_4_ crystal, respectively. The results demonstrated that the spinel structure of Fe_3_O_4_ did not undergo phase transformation during the grafting process, and Fe_3_O_4_ particles were incorporated into the prepared polymers [31].

**Figure 6:**
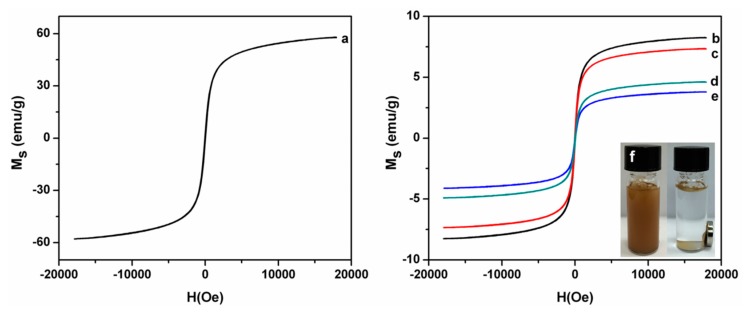
Magnetic hysteresis loops of the Fe_3_O_4_ particle (**a**), Fe_3_O_4_-MAH-β-CD@MIP (**b**), Fe_3_O_4_-MAH-β-CD@NIP (**c**), Fe_3_O_4_-CTs@MIP, (**d**) and Fe_3_O_4_-CTs@NIP (**e**).

The magnetic properties of the Fe_3_O_4_ particle, Fe_3_O_4_-MAH-β-CD@MIP, Fe_3_O_4_-MAH-β-CD@NIP, Fe_3_O_4_-CTs@MIP and Fe_3_O_4_-CTs@NIP were investigated by VSM as shown in Figure 6. It was clear that there was no hysteresis, and the symmetrical pass through the origin in the magnetization curves, indicates that all these magnetic particles retained super-paramagnetic properties. The saturation magnetization values (Ms) were 57.8 emu·g^−1^, 8.2 emu·g^−1^, 7.3 emu·g^−1^, 4.6 emu·g^−1^ and 3.8 emu·g^−1^. Compared with the pure Fe_3_O_4_, the saturation magnetization of polymers decreased significantly, which might be due to the decrease in the ratio of magnetic substances after the polymerization. However, Fe_3_O_4_-CTs@MIP and Fe_3_O_4_-MAH-β-CD@MIP can be separated rapidly from suspensions under an external magnetic field. The magnetic adsorbents adhered to the walls of the vial, and the supernatant became clear and transparent as shown in the inserted photograph marked with the letter “f”.

The weight percentages of Fe_3_O_4_ that adhered to Fe_3_O_4_-CTs@MIP, Fe_3_O_4_-CTs@NIP, Fe_3_O_4_-MAH-β-CD@MIP and Fe_3_O_4_-MAH-β-CD@NIP were measured by TGA as shown in Figure S2. Nearly 4.0% weight loss was observed at the range of 30 °C to 700 °C for the Fe_3_O_4_ particle, which could be due to the evaporation of absorbed water as well as dehydration of the surface –OH groups. There was no significant weight loss for other polymers under 200 °C, which could be due to the stability of the polymer. The weight of these imprinted polymers decreased significantly between 300 °C and 600 °C, which might be attributed to the decomposition of carbon skeleton. Finally, there was no weight loss above 600 °C. These results demonstrated that the magnetite contents of Fe_3_O_4_-MAH-β-CD@MIPs, Fe_3_O_4_-MAH-β-CD@NIPs, Fe_3_O_4_-CTs@MIP and Fe_3_O_4_-CTs@NIP were 21.1%, 19.1%, 17.3% and 14.6%, respectively, which were consistent with the saturation magnetization value.

### 3.3. Adsorption Study

#### 3.3.1. Effect of Solution pH on Adsorption

**Figure 7:**
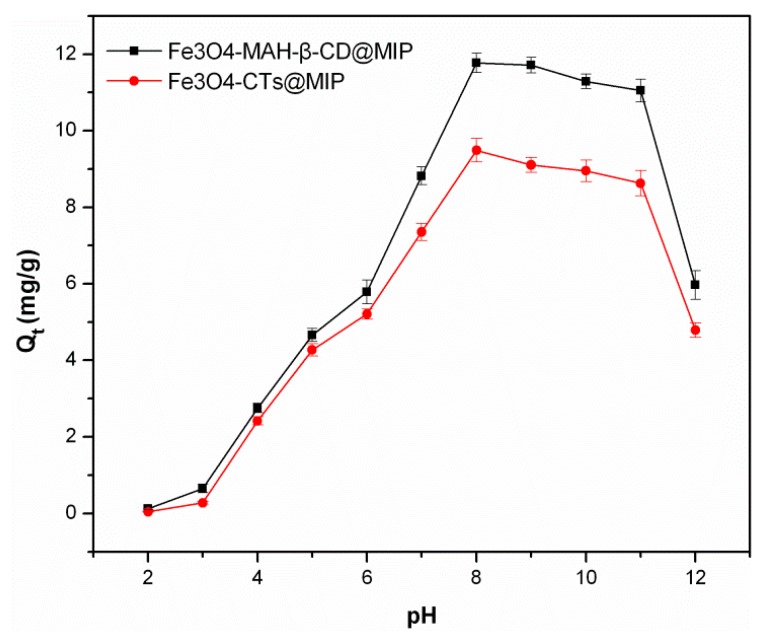
Effect of pH on the adsorption of 2-aminopyridine on the Fe_3_O_4_-MAH-β-CD@MIP and Fe_3_O_4_-CTs@MIP.

The effect of solution pH was investigated with the pH values ranging from 2.0 to 12.0, and the results are shown in Figure 7. It is clearly observed that the pH value had a great influence on the adsorption of 2-aminopyridine for the Fe_3_O_4_-CTs@MIP and Fe_3_O_4_-MAH-β-CD@MIP. The compound 2-aminopyridine has two nitrogen atoms; each has lone pair electrons to donate, but the ring nitrogen atom is known to be more basic in comparison with amino nitrogen. Based on the pKa value of 2-aminopyridine (pKa = 6.86), in a low pH solution, the ring nitrogen atom of 2-aminopyridine and amino group on the surface of Fe_3_O_4_-CTs@MIP is easily protonated, resulting in the fact that the 2-aminopyridine and adsorption sites of Fe_3_O_4_-CTs@MIP carried positive charges. The strong electrostatic repulsion makes the adsorption weak. This behavior is consistent with previous reports [32,33]. In addition, the carboxyl groups grafted on Fe_3_O_4_-MAH-β-CD@MIP rarely dissociate under a low pH condition. In this case, the electrostatic interaction between polymers and 2-aminopyridine is quite weak, resulting in low adsorption ability. Therefore, they have similar weak adsorption behavior at low pH. With the increase in pH, the dissociation degree of the carboxyl groups increases, making the electrostatic repulsion weaker and electrostatic interaction stronger. Meanwhile, the carboxyl group on the surface of Fe_3_O_4_-MAH-β-CD@MIP can form hydrogen bonds with amino nitrogen of aminopyridine, which makes more binding sites available. Thus, more 2-aminopyridine could be adsorbed onto the imprinted polymers. With the help of two interactions, the adsorption capacity rises rapidly when the solution pH is in a range of 6–8. The uptake of 2-aminopyridine onto Fe_3_O_4_-CTs@MIP may mainly contribute to hydrogen bonding between the hydroxyl group in chitosan and the amino group in 2-aminopyridine. The experimental results proved that the interaction between 2-aminopyridine and Fe_3_O_4_-CTs@MIP was slightly lower than that of Fe_3_O_4_-MAH-β-CD@MIP. When pH > 8, the weakening effect of the electrostatic interaction between polymers and 2-aminopyridine was displayed obviously, leading to a lower adsorption capacity. Therefore, the optimal pH value of 8 was chosen for subsequent adsorption studies.

#### 3.3.2. Adsorption Isotherm

The Langmuir [34] and Freundlich isotherm models [35], which can be expressed as Equations (2) and (3), respectively, are widely adopted for adsorption equilibrium data analysis.

(2) $$Q_{e}=\frac{K_{L}Q_{m}C_{e}}{1+K_{L}C_{e}}$$

(3) $$Q_{e}=K_{F}C_{e}{}^{1/n}$$

where Q_e_ (mg·g^−1^) is the equilibrium adsorption capacity, C_e_ (mg·mL^−1^) is the equilibrium concentration of 2-aminopyridine in aqueous solution, Q_m_ (mg·g^−1^) is the maximum adsorption capacity. K_L_ represents the Langmuir constant, K_F_ is the Freundlich constant and 1/n is the heterogeneity factor.

**Figure 8:**
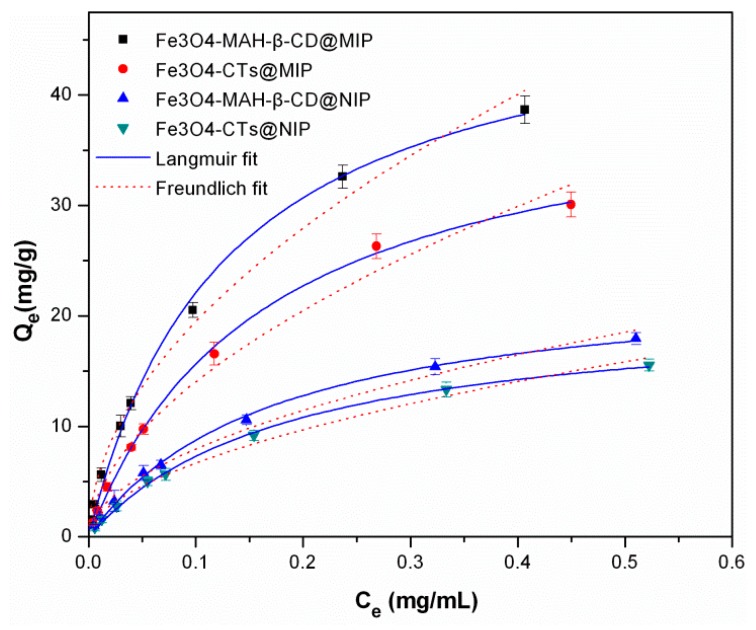
Adsorption isotherms of 2-aminopyridine on Fe_3_O_4_-CTs@MIP, Fe_3_O_4_-CTs@NIP, Fe_3_O_4_-MAH-β-CD@MIP and Fe_3_O_4_-MAH-β-CD@NIP at 298 K. (mean ± SD, n = 3).

The adsorption amounts of 2-aminopyridine versus the corresponding equilibrium concentration at 298 K were plotted as adsorption isotherms in Figure 8. With increasing 2-aminopyridine concentration, the adsorption capacity of 2-aminopyridine was increased clearly. The calculated isotherm parameters accompanied with standard errors of Fe_3_O_4_-CTs@MIP, Fe_3_O_4_-CTs@NIP, Fe_3_O_4_-MAH-β-CD@MIP and Fe_3_O_4_-MAH-β-CD@NIP for 2-aminopyridine adsorption was summarized in Table 1. Standard errors of three experiment replicates are within the range from 2.8 to 6.4%. By comparing the regression coefficients (R^2^), the Langmuir model fitted the data better compared to the Freundlich model. This indicated that adsorption might occur on the homogeneity active sites of the absorbent surface. The maximum adsorption capacities of 2-aminopyridine binding to Fe_3_O_4_-CTs@MIP and Fe_3_O_4_-MAH-β-CD@MIP calculated by the Langmuir isotherm model were 39.2 mg·g^−1^ and 46.5 mg·g^−1^, respectively, which were higher than those on the Fe_3_O_4_-CTs@NIP and Fe_3_O_4_-MAH-β-CD@NIP. This revealed that the synthesized MMIP had more specific sites than the MNIP for the template molecules.

The adsorption amount of 2-aminopyridine on Fe_3_O_4_-MAH-β-CD@MIP was higher than Fe_3_O_4_-CTs@MIP, which could be due to more hydrogen bonding and electrostatic interaction between Fe_3_O_4_-MAH-β-CD@MIP and 2-aminopyridine molecules. Significantly, the adsorption capacities of all the synthesized materials in this work were much higher than those of the previously reported conventional imprinted polymers [16,27]. However, the MIH-FRP hydrogel with high adsorption capacity of 3-aminopyridine was prepared in aqueous media, bringing a stronger electrostatic interaction between the template and monomer self-assembly. this provides a new approach for the preparation of high performance imprinted hydrogel [36]. In our work, MMIPs were prepared in organic solvents through imprinting technology based on novel supporting materials. They have promising imprinting inside the MMIPs network that has strong hydrogen bonding and electrostatic interaction between 2-aminopyridine and polymers. Moreover, the MMIPs are magnetic and can be separated rapidly from suspensions under an external magnetic field. The comparative results are shown in Table 2. To evaluate the reproducibility of MMIP preparation, the adsorption properties of three batches of MMIP were investigated. Table 3 shows the batch reproducibility data of Fe_3_O_4_-CTs@MIP and Fe_3_O_4_-MAH-β-CD@MIP. The results reveal that Fe_3_O_4_-CTs@MIP and Fe_3_O_4_-MAH-β-CD@MIP for 2-aminopyridine could be prepared consistently.

In addition, temperature as an important parameter may have an impact on the adsorption for 2-aminopyridine on Fe_3_O_4_-CTs@MIP and Fe_3_O_4_-MAH-β-CD@MIP. The adsorption isotherms of Fe_3_O_4_-CTs@MIP and Fe_3_O_4_-MAH-β-CD@MIP at various temperatures are illustrated in Figure S3. The isotherm parameters of 2-aminopyridine binding on Fe_3_O_4_-CTs@MIP and Fe_3_O_4_-MAH-β-CD@MIP at different temperatures are presented in Table 4. Clearly, one finds that the adsorption of 2-aminopyridine decreased as the temperature increased. The Langmuir equilibrium constant (K_L_) decreased with increasing temperature, indicating that the binding ability of 2-aminopyridine on Fe_3_O_4_-CTs@MIP and Fe_3_O_4_-MAH-β-CD@MIP was higher at lower temperature. Furthermore, the calculated Langmuir maximum adsorption capacities of Fe_3_O_4_-MAH-β-CD@MIP for 2-aminopyridine were higher than those of Fe_3_O_4_-CTs@MIP regardless of the experimental temperature.

### 3.4. Adsorption Kinetics

**Figure 9:**
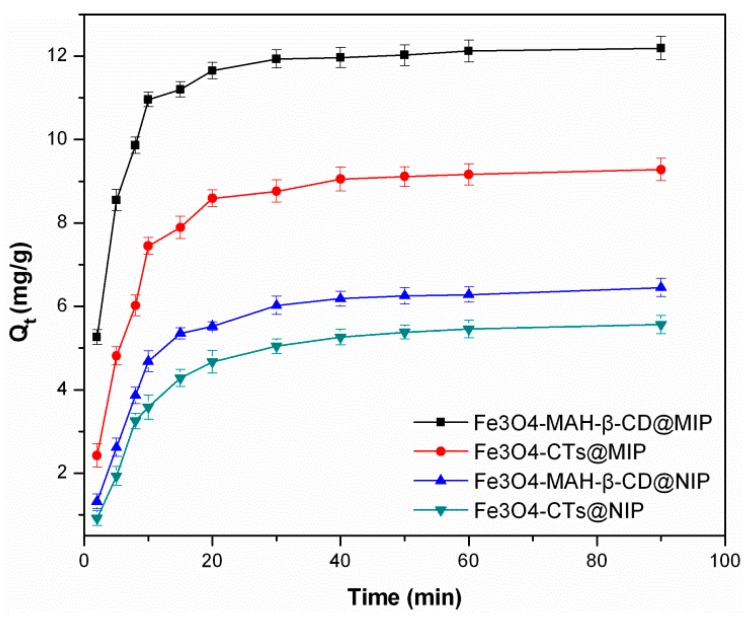
Adsorption kinetics curves of 2-aminopyridine on the Fe_3_O_4_-CTs@MIP, Fe_3_O_4_-CTs@NIP, Fe_3_O_4_-MAH-β-CD@MIP and Fe_3_O_4_-MAH-β-CD@NIP.

Figure 9 shows a time-dependent adsorption dynamics of 2-aminopyridine on Fe_3_O_4_-CTs@MIP, Fe_3_O_4_-MAH-β-CD@MIP and MNIPs at optimized pH and room temperature. The adsorption of all these four adsorbents was fast in the first 20 min and then achieved equilibrium after approximately 30 min. This was mainly due to the existence of a large number of active sites on the surface of adsorbents at the beginning. However, with the increase in the contact time, the slope of the adsorption capacity increased slowly, which might be attributed to the gradual occupation of the binding sites on the adsorbent surface by aminopyridine, resulting in decreased adsorption rate at the later stage. In addition, it was observed that the capacity of the adsorbed 2-aminopyridine on Fe_3_O_4_-CTs@MIP and Fe_3_O_4_-MAH-β-CD@MIP at any time was considerably greater than that of the corresponding MNIPs. This was mainly due to the specific recognition effect and affinity binding sites in imprinted polymers. The pseudo-first order [37] and pseudo-second order models [38] were used to evaluate the adsorption kinetics, which are described in the following Equations (4) and (5), respectively:

(4) $$\ln(Q_{e}-Q_{t})={\mathrm{lnQ}}_{e}-k_{1}t$$

(5) $$\frac{t}{Q_{t}}=\frac{1}{k_{2}Q_{e}^{2}}+\frac{t}{Q_{e}}$$

where k_1_ (min^−1^) and k_2_ (g·mg^−1^·min^−1^) are the rate constant of pseudo-first order and pseudo-second order adsorption, respectively; Q_e_ (mg·g^−1^) and Q_t_ (mg·g^−1^) are the adsorption capacity at equilibrium and time t, respectively.

The parameters of adsorption kinetics are summarized in Table S1. The regression curves of the pseudo-first-order and pseudo-second-order rate equation of linear forms for 2-aminopyridine adsorption are shown in Figures S4 and S5. It is known that the pseudo-second order kinetic model is assumed for the chemical reaction mechanisms, and that the adsorption rate is controlled by chemical adsorption between chemical binding sites on the surface of adsorbent and adsorbate. By comparison of the data, the adsorption processes of 2-aminopyridine on adsorbents were better fitted by the pseudo-second order model with a good correlation coefficient (R^2^ > 0.99). Therefore, the adsorption behavior of 2-aminopyridine onto adsorbents belonged to the pseudo-second order model, and the adsorption process was mainly a chemical process. The adsorption rate constant k_2_ for 2-aminopyridine on Fe_3_O_4_-MAH-β-CD@MIP was greater than that on Fe_3_O_4_-CTs@MIP, indicating a higher rate for 2-aminopyridine removal by Fe_3_O_4_-MAH-β-CD@MIP. Furthermore, compared with the pseudo-first order model, the calculated Q values (Q_e_, cal) obtained by the pseudo-second order model were highly consistent with the experimental data.

**Figure 10:**
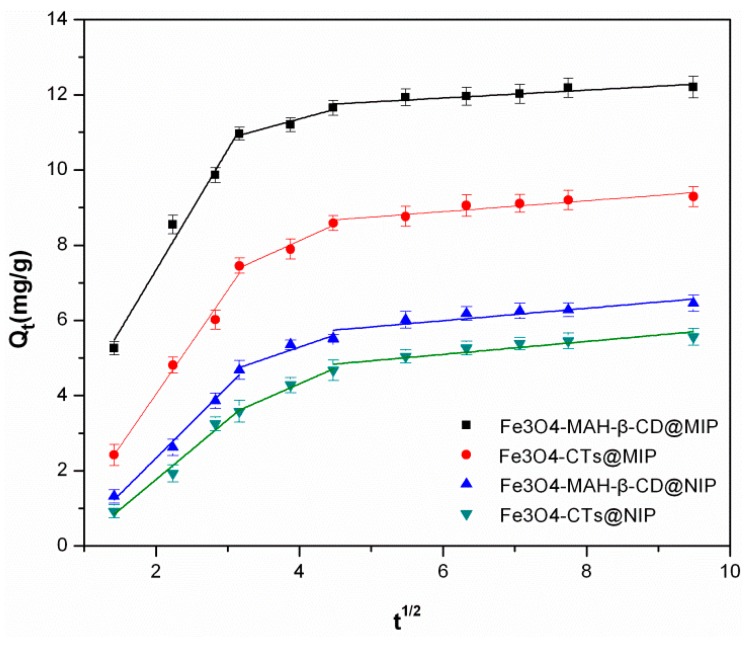
Intraparticle diffusion model for 2-aminopyridine adsorption onto Fe_3_O_4_-CTs@MIP, Fe_3_O_4_-CTs@NIP, Fe_3_O_4_-MAH-β-CD@MIP and Fe_3_O_4_-MAH-β-CD@NIP.

The intraparticle diffusion model was proposed for the interpretation of adsorption progress of aminopyridine intraparticle of the imprinted polymers. Equation (6) is given by Weber and Morris [39] as below:

(6) $$Q_{t}=k_{i}t^{1/2}+C$$

where C (mg·g^−1^) is a constant which reflects the thickness of the boundary layer and k_i_ is the intra-particle diffusion rate constant (mg·g^−1^·min^−1/2^). If the curve of Q_t_ against t^1/2^ passes through the origin, the adsorption process is only controlled by intra-particle diffusion. Otherwise, some other mechanisms may be involved in the adsorption process. Curves of Q_t_ against t^1/2^ for 2-aminopyridine adsorption onto absorbents are presented in Figure 10. It exhibited three linear plots for 2-aminopyridine adsorption, indicating three steps influenced in the adsorption process. In the initial fast step, the diffusion of 2-aminopyridine may cause the rapid adsorption. Then, intraparticle diffusion into mesopores and micropores in the gradual adsorption stage is the rate determining step. In the final stage, the slowdown of intraparticle diffusion may be attributed to the relatively low content of residual 2-aminopyridine in the solution [40,41]. Therefore, the intraparticle diffusion was involved in the adsorption process but was not the only rate determining step.

### 3.5. Adsorption Thermodynamics

Figure S6 shows that, as the temperature increased, the values of ln(C_a_/C_e_) decreased, illustrating the exothermic nature of the 2-aminopyridine adsorption process. The values of ln(C_a_/C_e_) at various temperatures were calculated using Equation (7) as proposed by Van’t Hoff [42]:

(7) $$\ln\;\left(\frac{C_{a}}{C_{e}}\right)=-\frac{\Delta H}{\left(\mathrm{RT}\right)}+\frac{\Delta S}{R}$$

The Gibbs free energy Δ*G* (kJ·mol^−1^) and entropy change Δ*S* of adsorption are described by Equations (8) and (9), respectively, as follows [43]:

(8) $$\Delta G=-RT\ln\;\left(\frac{C_{a}}{C_{e}}\right)$$

(9) $$\Delta S=\frac{\Delta H-\Delta G}{T}$$

where, C_a_ and C_e_ are the equilibrium concentrations of 2-aminopyridine on the adsorbents and solution at different temperatures, respectively. R is the universal gas constant (8.314 J/mol·K) and *T* is the absolute temperature (K). The enthalpy change (Δ*H*, kJ·mol^−1^) was calculated from the slope of the line plotted by ln(C_a_/C_e_) against 1/*T*. The calculated thermodynamic parameters are presented in Table 5. It was clearly observed that the values of Δ*G* were negative for both the adsorption process on Fe_3_O_4_-CTs@MIP and Fe_3_O_4_-MAH-β-CD@MIP at various temperatures, illustrating that the adsorption process on the two adsorbents towards 2-aminopyridine were spontaneous. In addition, the observed negative values of Δ*G* decreased as the temperature increased, indicating that the higher temperature has an adverse effect on the adsorption. The negative values of entropy Δ*S* suggested the decreased randomness at the solid-liquid interface during the adsorption of 2-aminopyridine onto both absorbents. Finally, the obtained negative ΔH values also further confirmed the exothermic nature of the adsorption process [44].

### 3.6. Selective Adsorption

**Figure 11:**
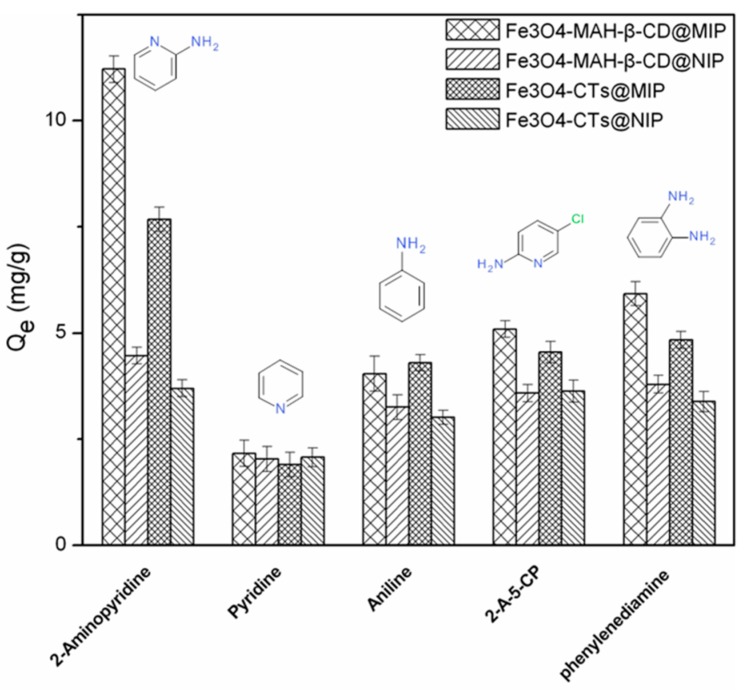
Binding selectivity of Fe_3_O_4_-CTs@MIP and Fe_3_O_4_-MAH-β-CD@MIP.

The selective adsorption performances of Fe_3_O_4_-CTs@MIP, Fe_3_O_4_-CTs@NIP, Fe_3_O_4_-MAH-β-CD@MIP and Fe_3_O_4_-CTs@NIP were investigated by comparison of adsorption capacities of 2-aminopyridine and their structural analogues including pyridine, aniline, 2-amino-5-chloropyridine (2-A-5-CP), phenylenediamine. The adsorption experiments were performed independently by adding one of these polymers into the solution of each analogue with shaking for 24 h. Figure 11 shows the adsorption selectivity of MMIP and MNIP for 2-aminopyridine and its analogues. The results demonstrated that all the MMIPs have higher selective affinity for 2-aminopyridine and its analogues compared with that of MNIPs except for pyridine. The differences between MMIPs and MNIPs are due to the recognition effect of MMIPs. The exception for pyridine can be explained by its structure: there was no substitute group on the ring. The result also showed that both Fe_3_O_4_-MAH-β-CD@MIP and Fe_3_O_4_-CTs@MIP exhibited much higher selectivity for 2-aminopyridine than those of the other four analogs. The reason can be attributed to the effect of imprinting with 2-aminopyridine, which is a template of monosubstituted pyridine. It is clearly suggests the presence of specific imprinting sites that favored the adsorption of the target molecule. For di-substituted pyridine 2-A-5-CP and aminobenzene compounds of aniline and phenylenediamine, the relative lower adsorption amounts may also be related to the steric hindrance of the two substitute groups and the ring structure without nitrogen. Therefore, it can be indicated that Fe_3_O_4_-CTs@MIP and Fe_3_O_4_-MAH-β-CD@MIP have good selectivity and specific recognition toward 2-aminopyridine due to the imprinting effect, even with the interference of competitive analogues.

### 3.7. Influence of Coexistent Ions

**Figure 12:**
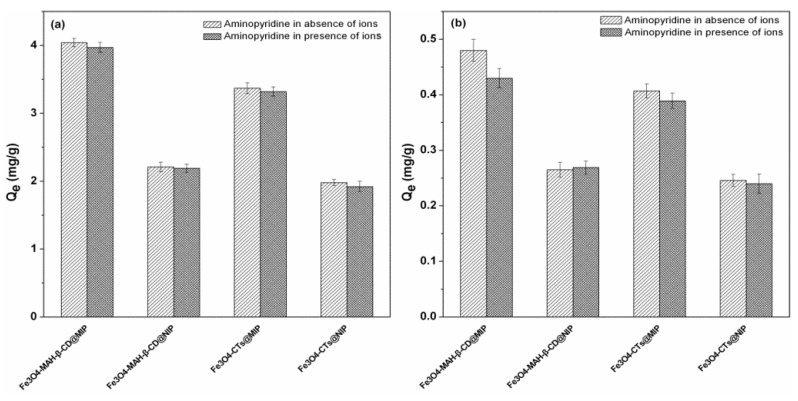
Influence of coexisting ions on the aminopyridine binding onto Fe_3_O_4_-CTs@MIP, Fe_3_O_4_-MAH-β-CD@MIP and MNIPs. (**a**) C_0,aminopyridine_: 0.01 mg·mL^−1^; (**b**) C_0,aminopyridine_: 0.001 mg·mL^−1^, C_Na_^+^: 1.149 mg·mL^−1^, C_K_^+^: 0.039 mg·mL^−1^, C_Mg_^2+^: 0.122 mg·mL^−1^, C_Ca_^2+^: 0.04 mg·mL^−1^, C_Cl_^−^: 1.882 mg·mL^−1^, C_SO4_^2−^: 0.48 mg·mL^−1^.

In the presence of coexistent ions including Na^+^, K^+^, Mg^2+^, Ca^2+^, Cl^−^ and SO_4_^2−^ in water samples, the binding efficiency and selective ability of Fe_3_O_4_-CTs@MIP, Fe_3_O_4_-CTs@NIP, Fe_3_O_4_-MAH-β-CD@MIP and Fe_3_O_4_-MAH-β-CD@NIP for 2-aminopyridine were investigated and the results are presented in Figure 12. It is observed that the binding capacity of synthesized MMIPs and MNIPs shows no significant change in both the absence and presence of various ions in water samples. This means that the binding was hardly influenced by the coexisting ions in water. Thus, the MMIPs synthesized in our work were possibly involved in detecting or removing 2-aminopyridine in water.

### 3.8. Comparison of MIPs and Activated Carbon in Selective Adsorption Performance

**Figure 13:**
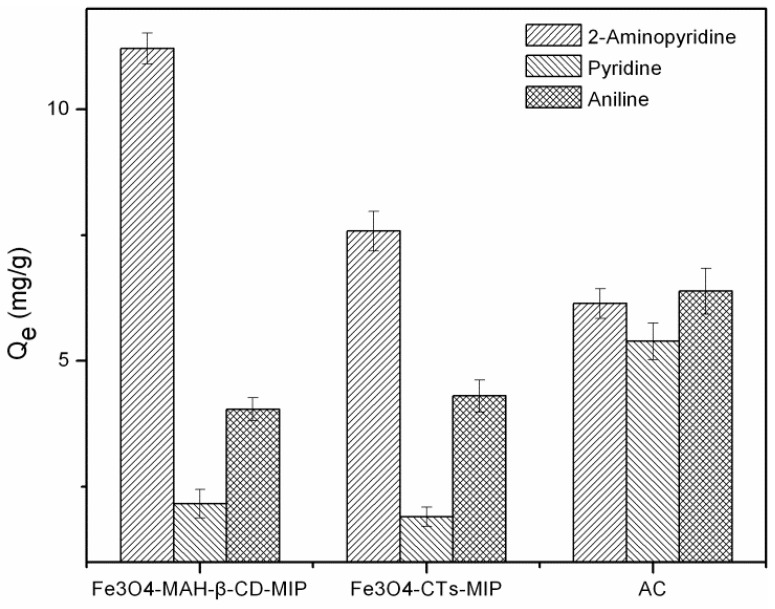
Selective adsorption performance of spiked water with different sorbents.

For comparison, the adsorption and selective performance of the synthesized MMIPs were compared with those of commercial granular activated carbon (GAC) for the treatment of 2-aminopyridine and analogues in water. GAC is activated carbon with particle sizes predominantly greater than 80 mesh and a surface area between 900 and 1110 m^2^·g^−1^. As shown in Figure 13, owing to the imprinting effect, two MMIPs showed a higher recognition and selective ability to 2-aminopyridine compared with activated carbon. The Fe_3_O_4_-MAH-β-CD@MIP exhibited the greatest affinity among the three adsorbents. The commercial activated carbon material showed a similar adsorption ability to all three organic substances, and the selectivity was not obvious.

### 3.9. Regeneration and Reusable Studies

**Figure 14:**
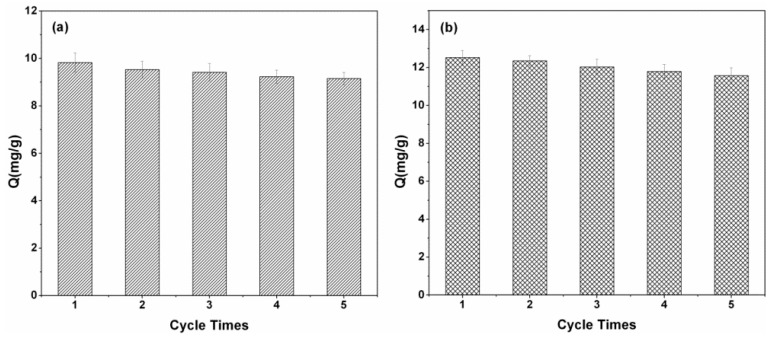
Regeneration experiments for (**a**) Fe_3_O_4_-CTs@MIP and (**b**) Fe_3_O_4_-MAH-β-CD@MIP.

The regeneration properties of Fe_3_O_4_-CTs@MIP and Fe_3_O_4_-MAH-β-CD@MIP after adsorption were investigated to understand the possibility of further practical application. As shown in Figure 14, the synthesized MMIPs can be regenerated after washing with methanol/acetic acid (90:10, *v/v*). Their adsorption efficiencies were stable for up to five adsorption—regeneration cycles without a considerable decrease for 2-aminopyridine. After the fifth regeneration, the reduction in the adsorption capacity of Fe_3_O_4_-CTs@MIP and Fe_3_O_4_-MAH-β-CD@MIP for 2-aminopyridine was only 6.9% and 7.6%, respectively. The result indicated that Fe_3_O_4_-CTs@MIP and Fe_3_O_4_-MAH-β-CD@MIP are desirable for potential application in future.

### 3.10. Removal of 2-Aminopyridine with MMIPs in Different Water Samples

For evaluating the possibility of practical applications, the adsorption performance of the synthesized MMIPs for the genotoxic compound 2-aminopyridine in different water samples was investigated. Usually, for the discharged water after routine treatment, the level of the pollutant may be about 1–10 mg·L^−1^. Therefore, the experimental water samples of spiked 10 mg·L^−1^ 2-aminopyridine in deionized water, tap water and river water samples were used for evaluation.

**Figure 15:**
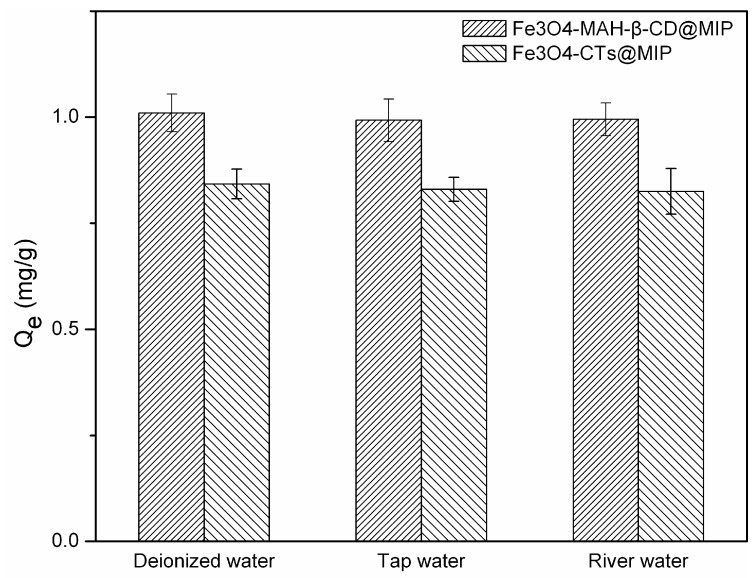
Removal of 2-aminopyridine from different types of real water samples.

As shown in Figure 15, there was no significant difference for the adsorption amount of synthesized MMIP for 2-aminopyridine in deionized water, tap water and real river water samples. The results indicated that, owing to the existence of specific binding sites in Fe_3_O_4_-CTs@MIP and Fe_3_O_4_-MAH-β-CD@MIP, the MMIPs have a good adsorption performance for 2-aminopyridine with high selectivity and efficiency in all kinds of experimental water samples.

## 4. Conclusions

In this study, two novel magnetic molecular imprinted polymers based on chitosan and MAH-β-CD were successfully synthesized by a molecular imprinting technique for the selective removal and magnetic separation of 2-aminopyridine in aqueous solutions. Adsorption experimental results showed that the synthesized MMIPs materials had high adsorption capacity and good selectivity for 2-aminopyridine owing to the imprinting effect. Compared with other sorbents reported in the literature, the adsorption performance of MIPs prepared in this work showed a significant improvement. The adsorption capacity of Fe_3_O_4_-MAH-β-CD@MIP was higher than that of Fe_3_O_4_-CTs@MIP, which could be attributed to the stronger action on hydrogen bonding and electrostatic attraction between Fe_3_O_4_-MAH-β-CD@MIP and the 2-aminopyridine target molecule. In addition, the synthesized MMIPs exhibited higher specific recognition and selectivity to 2-aminopyridine in the presence of interference substances and showed excellent performance of regeneration. It can be used at least five times with little adsorption capacity loss. Therefore, the imprinted polymers are expected to have promising application for the removal and analysis of 2-aminopyridine in environmental water. However, in order to have a larger scale and practical application in future, numerous different factors should be considered, and more work is required.

## Figures and Tables

**Table 1:** Adsorption isotherm parameters for 2-aminopyridine on Fe_3_O_4_-CTs@MIP, Fe_3_O_4_-CTs@NIP, Fe_3_O_4_-MAH-β-CD@MIP and Fe_3_O_4_-MAH-β-CD@NIP (mean ± SD, n = 3).

Polymer	Langmuir Isotherm		R^2^	Freundlich Isotherm		R^2^
	Q_m_ (mg·g^−1^)	K_L_ (mL·mg^−1^)		K_F_ (mg^1-1/n^·mL^1/n^·g^−1^)	1/n	
Fe_3_O_4_-CTs@MIP	39.22 ± 1.466	7.29 ± 0.682	0.998	64.22 ± 3.316	0.678 ± 0.0392	0.981
Fe_3_O_4_-CTs@NIP	19.88 ± 0.577	6.29 ± 0.414	0.998	28.51 ± 1.353	0.655 ± 0.0326	0.987
Fe_3_O_4_-MAH-β-CD@MIP	46.51 ± 1.314	10.24 ± 0.634	0.996	81.85 ± 3.797	0.629 ± 0.0277	0.989
Fe_3_O_4_-MAH-β-CD@NIP	22.52 ± 0.823	6.94 ± 0.483	0.997	33.11 ± 2.113	0.641 ± 0.0468	0.987

**Table 2:** Comparison of the maximum adsorption capacity of aminopyridine on prepared MMIPs with other reported adsorbents.

Adsorbents	Target	Q_m_ (mg·g^−1^)	References
Bulk polymerized imprinted polymer	2-aminopyridine	12.8	[16]
Hybrid MIP with NIPA hydrogel	4-aminopyridine	6.0	[27]
MIH-FRP hydrogel	3-aminopyridine	600.0	[36]
Magnetic chitosan-based imprinted polymer	2-aminopyridine	39.2	This study
Magnetic β-cyclodextrin based imprinted polymer	2-aminopyridine	46.5	This study

**Table 3:** Batch reproducibility of Fe_3_O_4_-CTs@MIP and Fe_3_O_4_-MAH-β-CD@MIP *.

MIPs	Adsorption Capacity (Q_e_, mg·g^−1^) of Fe_3_O_4_-CTs@MIP	Adsorption Capacity (Q_e_, mg·g^−1^) of Fe_3_O_4_-MAH-β-CD@MIP
Batch#1	9.28	12.19
Batch#2	10.04	12.65
Batch#3	8.71	14.06
Average ± SD	9.34 ± 0.657	12.97 ± 0.978

* Adsorption of 2-aminopyridine on the Fe_3_O_4_-CTs@MIP and Fe_3_O_4_-MAH-β-CD@MIP at 298 K; the initial concentration of 2-aminopyridine was 0.1 mg·mL^−1^; the dosage of adsorbents was 2 mg·mL^−^^1^.

**Table 4:** Adsorption isotherm parameters for 2-aminopyridine on Fe_3_O_4_-CTs@MIP and Fe_3_O_4_-MAH-β-CD@MIP at various temperatures (mean ± SD, n = 3).

Adsorption Isotherm Models	Constants	Fe_3_O_4_-CTs@MIP					Fe_3_O_4_-MAH-β-CD@MIP				
		288 K	298 K	308 K	318 K	328 K	288 K	298 K	308 K	318 K	328 K
Langmuir isotherm	Q_m_ (mg·g^−1^)	41.32 ± 1.917	39.22± 1.466	34.60 ± 0.977	30.68 ± 0.872	27.25 ± 0.967	48.78 ± 1.737	46.51 ± 1.374	41.67 ± 1.407	37.45 ± 1.139	33.56 ± 0.854
	K_L_ (mL·mg^−1^)	8.3 ± 0.591	7.29 ± 0.682	5.67 ± 0.375	4.87 ± 0.297	4.32 ± 0.268	12.81 ± 0.908	10.24 ± 0.634	7.27 ± 0.481	5.68 ± 0.339	4.89 ± 0.289
	R^2^	0.998	0.998	0.998	0.998	0.998	0.994	0.996	0.998	0.999	0.999
Freundlich isotherm	K_F_ (mg^1-1/n^·mL^1/n^·g^−1^)	69.86 ± 3.474	64.22 ± 3.316	53.06 ± 3.285	44.59 ± 2.701	37.82 ± 2.276	87.42 ± 3.474	81.85 ± 3.797	68.16 ± 4.349	57.29 ± 3.598	48.68 ± 2.887
	1/n	0.66 ± 0.0482	0.678 ± 0.0392	0.71 ± 0.0519	0.72 ± 0.0517	0.74 ± 0.0511	0.59 ± 0.0482	0.63 ± 0.0277	0.68 ± 0.0497	0.70 ± 0.0507	0.72 ± 0.0481
	R^2^	0.981	0.981	0.982	0.982	0.982	0.989	0.989	0.988	0.988	0.988

**Table 5:** Thermodynamic parameters for adsorption of 2-aminopyridine on Fe_3_O_4_-CTs@MIP and Fe_3_O_4_-MAH-β-CD@MIP at various temperatures.

Polymers	T (K)	Thermodynamic Parameters			R^2^
		ΔG (kJ·mol^−1^)	ΔH (kJ·mol^−1^)	ΔS (J·mol^−1^·k^−1^)	
Fe_3_O_4_-CTs@MIP	288	−2.81	−11.26	−29.31	0.991
	298	−2.64		−28.89	
	308	−2.23		−29.28	
	318	−1.94		−29.29	
	328	−1.70		−29.12	
Fe_3_O_4_-MAH-β-CD@MIP	288	−3.76	−16.14	−42.98	0.990
	298	−3.47		−42.50	
	308	−2.85		−43.15	
	318	−2.44		−43.08	
	328	−2.13		−42.69	

## Supplementary Materials

The following are available online at www.mdpi.com/1660-4601/14/9/991/s1, Figure S1: FT-IR spectra of MAH-β-CD (a); Fe_3_O_4_-CTs@MIP and Fe_3_O_4_-CTs@NIP (b); Fe_3_O_4_-MAH-β-CD@MIP and Fe_3_O_4_-MAH-β-CD@NIP (c); Figure S2: TGA curves of the Fe_3_O_4_ particle, Fe_3_O_4_-CTs@MIP, Fe_3_O_4_-CTs@NIP, Fe_3_O_4_-MAH-β-CD@MIP and Fe_3_O_4_-MAH-β-CD@NIP; Figure S3: Adsorption isotherms of 2-aminopyridine binding onto Fe_3_O_4_-CTs@MIP (a) and Fe_3_O_4_-MAH-β-CD@MIP (b) at different temperatures; Figure S4: Pseudo-first-order kinetic model for the adsorption of 2-aminopyridine adsorption on Fe_3_O_4_-CTs@MIP, Fe_3_O_4_-CTs@NIP, Fe_3_O_4_-MAH-β-CD@MIP and Fe_3_O_4_-MAH-β-CD@NIP; Figure S5: Pseudo-second-order kinetic model for the adsorption of 2-aminopyridine adsorption on Fe_3_O_4_-CTs@MIP, Fe_3_O_4_-CTs@NIP, Fe_3_O_4_-MAH-β-CD@MIP and Fe_3_O_4_-MAH-β-CD@NIP; Figure S6: Van’t Hoff plots of the uptake of 2-aminpyridine on Fe_3_O_4_-CTs@MIP and Fe_3_O_4_-MAH-β-CD@MIP; Table S1: Kinetic parameters of the pseudo-first-order and pseudo-second-order rate equations for 2-aminopyridine adsorption on Fe_3_O_4_-CTs@MIP, Fe_3_O_4_-CTs@NIP, Fe_3_O_4_-MAH-β-CD@MIP and Fe_3_O_4_-MAH-β-CD@NIP (mean ± SD, *n* = 3).
