# Supplementary material for: Selective Removal of the Genotoxic Compound 2-Aminopyridine in Water using Molecularly Imprinted Polymers Based on Magnetic Chitosan and β-Cyclodextrin
Source: Int J Environ Res Public Health. 2017 Aug 31;14(9):991. doi: 10.3390/ijerph14090991 (PMC5615528; doi:10.3390/ijerph14090991)
Supplement: Supplementary file 1 [file ijerph-14-00991-s001.pdf]

Supplementary Materials

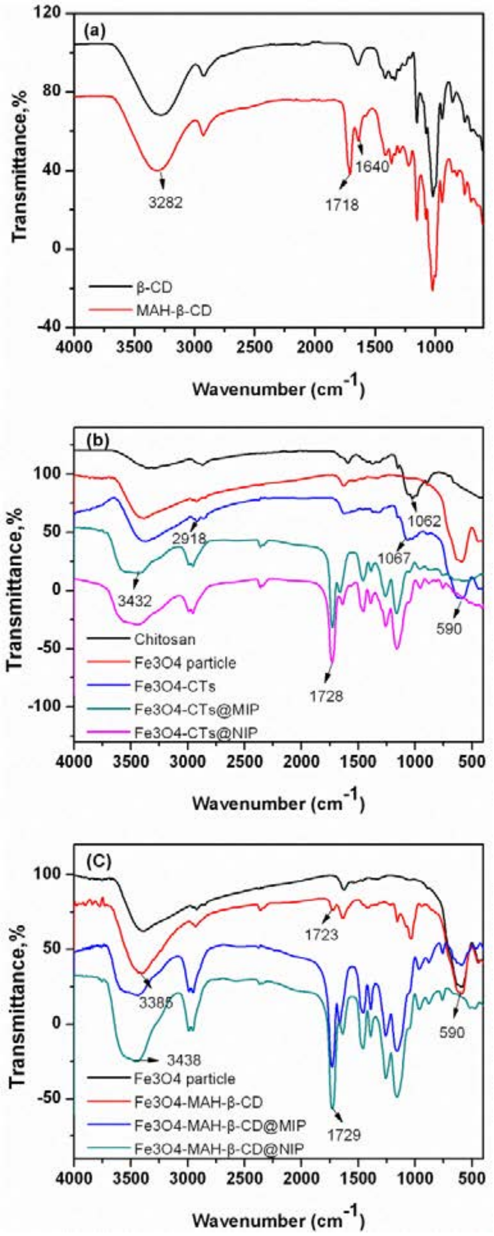

Figure S1. The FT-IR spectra of MAH-β-CD (**a**); Fe_3O_4-CTs@MIP and Fe_3O_4-CTs@NIP (**b**); Fe_3O_4-MAH-β-CD@MIP and Fe_3O_4-MAH-β-CD@NIP (**c**).

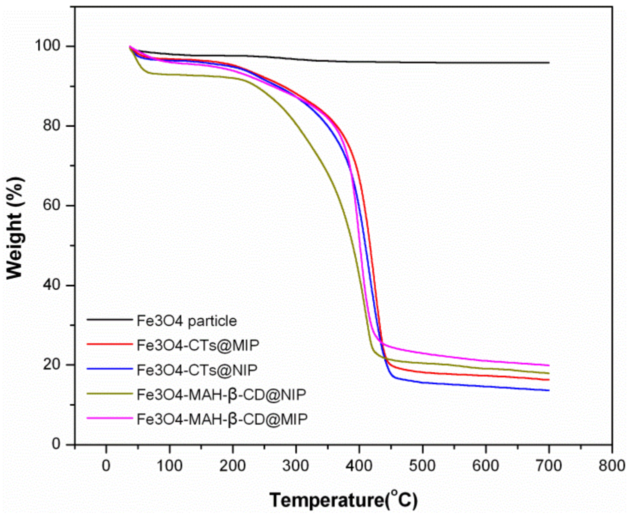

Figure S2. TGA curves of the Fe_3O_4 particle, Fe_3O_4-CTs@MIP, Fe_3O_4-CTs@NIP, Fe_3O_4-MAH-β-CD@MIP and Fe_3O_4-MAH-β-CD@NIP.

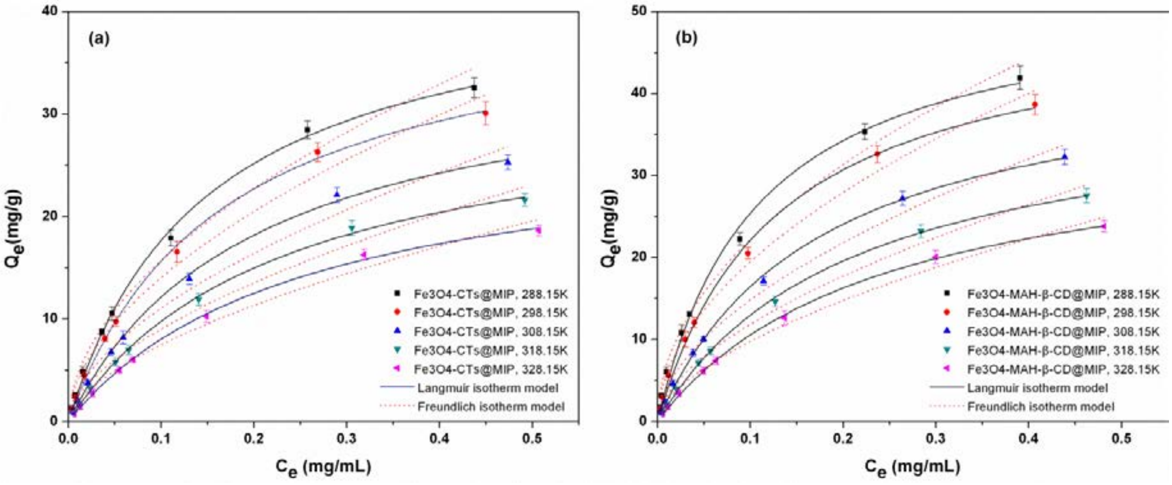

Figure S3. Adsorption isotherms of 2-aminopyridine binding onto Fe_3O_4-CTs@MIP (**a**) and Fe_3O_4-MAH-β-CD@MIP (**b**) at different temperatures.

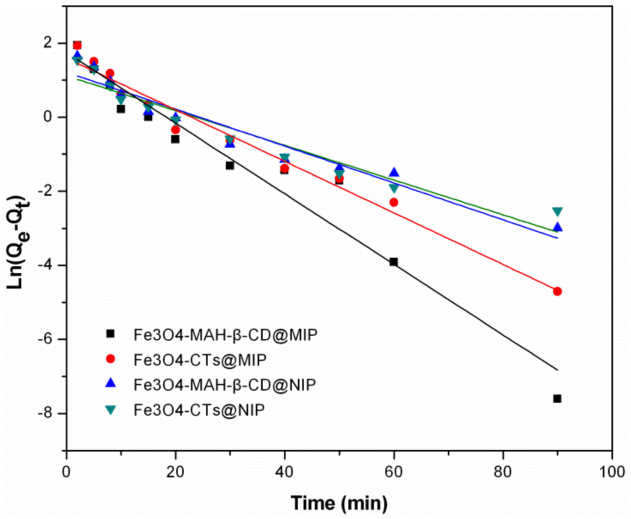

Figure S4. Pseudo-first-order kinetic model for the adsorption of 2-aminopyridine adsorption on Fe_3O_4-CTs@MIP, Fe_3O_4-CTs@NIP, Fe_3O_4-MAH-β-CD@MIP and Fe_3O_4-MAH-β-CD@NIP.

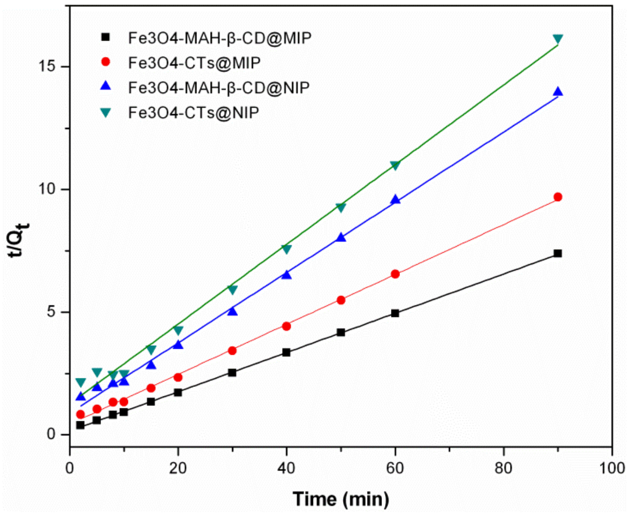

Figure S5. Pseudo-second-order kinetic model for the adsorption of 2-aminopyridine adsorption on Fe_3O_4-CTs@MIP, Fe_3O_4-CTs@NIP, Fe_3O_4-MAH-β-CD@MIP and Fe_3O_4-MAH-β-CD@NIP.

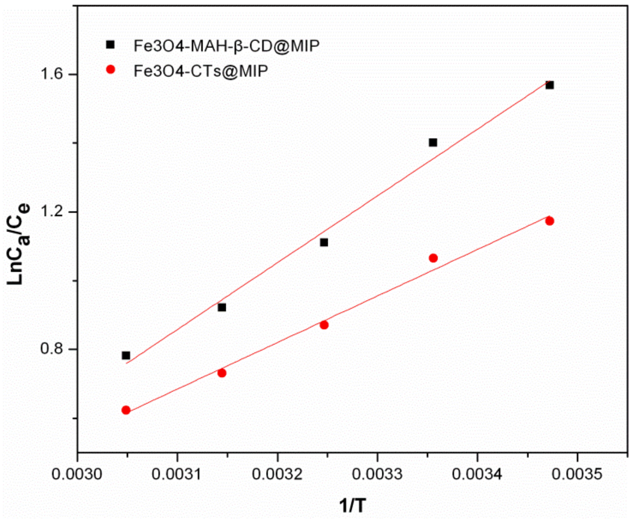

Figure S6. Van't Hoff plots of the uptake of 2-aminpyridine on Fe_3O_4-CTs@MIP and Fe_3O_4-MAH-β-CD@MIP.

Table S1. Kinetic parameters of the pseudo-first-order and pseudo-second-order rate equations for 2-aminopyridine adsorption on Fe_3O_4-CTs@MIP, Fe_3O_4-CTs@NIP, Fe_3O_4-MAH-β-CD@MIP and Fe_3O_4-MAH-β-CD@NIP (mean ± SD, *n*=3).

Polymer	Pseudo-First Order		R^2	Pseudo-Second Order		R^2
	k_1 (min^{-1})	$Q_{e,cal}$ (mg·g^{-1})		k_2 (g·mg^{-1}·min^{-1})	$Q_{e,cal}$ (mg·g^{-1})	
Fe_3O_4-CTs@MIP	0.0696	4.93	0.9731	0.0239	9.83	0.9995
Fe_3O_4-CTs@NIP	0.0469	3.05	0.9252	0.0208	6.15	0.9945
Fe_3O_4-MAH-β-CD@MIP	0.0953	5.71	0.9460	0.0416	12.50	0.9997
Fe_3O_4-MAH-β-CD@NIP	0.0498	3.37	0.9375	0.0232	6.98	0.9971
